# Characterization of the craniofacial abnormalities of the homozygous G608G progeria mouse model

**DOI:** 10.3389/fphys.2024.1481985

**Published:** 2024-11-06

**Authors:** Indeevar Beeram, Maria Belen Cubria, Pramod Kamalapathy, Diana Yeritsyan, Amanda J. Dubose, Ahmad Hedayatzadeh Razavi, Nazanin Nafisi, Michael R. Erdos, Brian D. Snyder, Wayne A. Cabral, Francis S. Collins, Ara Nazarian

**Affiliations:** ^1^ Musculoskeletal Translational Innovation Initiative, Carl J. Shapiro Department of Orthopaedic Surgery, Beth Israel Deaconess Medical Center, Harvard Medical School, Boston, MA, United States; ^2^ Molecular Genetics Section, Center for Precision Health Research, National Human Genome Research Institute, National Institutes of Health, Bethesda, MD, United States; ^3^ Department of Orthopaedic Surgery, Boston Children’s Hospital, Harvard Medical School, Boston, MA, United States; ^4^ Department of Orthopaedic Surgery, Yerevan State Medical University, Yerevan, Armenia

**Keywords:** progeria, homozygous G608G progeria mouse model, maxillofacial abnormality, microCT (μCT), morphology

## Abstract

**Introduction:**

Hutchinson-Gilford Progeria Syndrome (HGPS) is a rare genetic condition characterized by premature aging, impacting multiple organ systems, including cardiovascular, musculoskeletal, and integumentary. Significant abnormalities in a transgenic mouse model (homozygous G608G mutation), specifically targeting the development of skull and facial bone indices through high-resolution CT scanning and cephalometric analysis.

**Methods:**

Key measurements include bone thickness, skull volume, and cranial suture integrity. Bone volume increased significantly in HGPS mice by 8 months of age compared to wildtype mice.

**Results:**

Cortical thickness showed a trend toward increased values in HGPS mice. Cranial metrics revealed distinct differences.

**Discussion:**

HGPS mice exhibited smaller internasal width, interzygomatic distance, and palatine length compared to WT mice over time.

## Introduction

Hutchinson-Gilford Progeria Syndrome (HGPS) is a rare senescence syndrome affecting the cardiovascular, musculoskeletal (MSK), and integumentary systems. Although patients appear normal at birth, manifestations of HGPS appear within the first year of life, including growth deficiency, alopecia, loss of subdermal fat, and sclerodermatous-like changes to the skin (Ullrich et al., 2012). However, it is the rapidly progressive changes in the cardiovascular and cerebrovascular systems that lead to premature death during the second decade of life (Capell et al., 2008). Some of the most significant consequences of HGPS involving the MSK system include skeletal dysplasia with joint contractures, in addition to craniofacial abnormalities that become more evident around 2 years of age and progress with time (Merideth et al., 2008). These features include thinning of the calvarium, mottling of the skull in the frontal, parietal, and sphenoid regions, and widening cranial sutures. Other prevalent cranial abnormalities in HGPS include orbital hypotelorism, resulting in prominent eyes, a narrow nasal bridge with a broad-tipped nose, and a large skull relative to facial size (Ullrich et al., 2012). HGPS children also exhibit other craniofacial abnormalities, such as thin zygomatic arches and J-shaped sellas.

Most cases of HGPS are caused by a sporadic autosomal dominant mutation in the *LMNA* gene (c.1824C > T, p. G608G) (De Sandre-Giovannoli et al., 2003; Eriksson et al., 2003). Prelamin A, the precursor protein of Lamin A, undergoes normal post-translational processing of its C-terminal region. Sequential post-translational modifications include transient farnesylation of the cysteine at the C-terminal CaaX motif, proteolytic cleavage of the last three amino acids (aaX) by the metalloproteinase ZMPSTE24, and carboxymethylation. Finally, ZMPSTE24 cleaves the last 15 amino acids in the C-terminal region, removing the farnesyl group (Eriksson et al., 2003). These post-translational modifications lead to a mature, unfarnesylated Lamin A protein (Gargiuli et al., 2018; Harhouri et al., 2018), which localizes to the inner nuclear membrane and broadly influences nuclear structure and function (Broers et al., 2006). In HGPS, the G608G mutation activates a cryptic splice site, thereby deleting 50 amino acid residues containing the second endoproteolytic cleavage site and resulting in a truncated, permanently farnesylated protein termed “progerin” or LMNAΔ50 (Hernandez et al., 2010). Persistent farnesylation causes progerin accumulation at the inner nuclear membrane and difficulties reassembling the daughter nuclei properly after mitosis (Goldman et al., 2004).

Various murine models have been developed to mimic the bone abnormalities observed in Hutchinson-Gilford Progeria Syndrome (HGPS). The ZMPSTE24^−/−^ mouse was one of the first, displaying musculoskeletal (MSK) abnormalities such as kyphosis, spontaneous fractures by 6–7 weeks, and reduced zig-zag cranial sutures. This model mirrors some bone issues seen in progeria patients (Bergo et al., 2002). The Lmna^G609G/G609G^ mouse model was created recently, showing skeletal deformities such as lordo-kyphosis, joint immobility, and skull abnormalities (Osorio et al., 2012; Gargiuli et al., 2018). This model also highlights smaller lower incisors and delayed bone mineralization, including maxillary, mandibular, and calvarial structures. *In vitro* and *in vivo* studies of these mice reveal altered differentiation of bone cell populations and post-natal bone loss linked to defective Wnt signaling and reduced osteoclast activity (Cabral et al., 2023). The progression and characteristics of bone abnormalities in these murine models offer valuable insights into the mechanisms underlying HGPS and may guide therapeutic strategies to manage bone dysplasia in affected patients.

We have previously reported the generation of our transgenic mouse model carrying a human BAC harboring the common HGPS dominant-negative mutation (*LMNA* ^G608G^), which makes it possible to test novel therapeutic approaches in the context of the human gene, RNA, and protein sequences. Whereas single-copy transgenic mice (*LMNA* ^G/+^) do not develop pathologic changes outside of the vascular system before 20 months of age, double-copy mice faithfully recapitulate the vascular, dermal, and musculoskeletal phenotypic features of HGPS in just a few months (Varga et al., 2006; Cabral et al., 2021). A general phenotypic description by Cabral *et al.* notes reduced femoral trabecular bone volume (Tb BV/TV) by 5 months of age; a more detailed characterization of the long bone phenotype in *LMNA* ^G608G/G608G^ mice has been published by Cubria *et al.* Specifically, the authors noted reduced cortical thickness (Ct Th) with normal BMD and no significant difference in trabecular volume (Tb BV/TV) compared to age-matched wild-type mice. The different findings by Cabral et al. and Cubria et al. in the same mouse model may be due to a difference in the number of mice analyzed and the age of the mice (5 months *versus* 8 months/endpoint). However, the higher SMI in the trabecular bone of *LMNA* ^G/G^ mice suggested a more rod-like structure that is characteristic of normally aged bone. Furthermore, femoral head articular cartilage volume and GAG content were significantly decreased, as occurs in the early stages of osteoarthritis. These previous studies did not, however, focus on the cranial bones.

Inasmuch, no mouse model has accurately reproduced the craniofacial changes found in humans with HGPS to test new therapeutic strategies and their potential impact on the natural course of the disease. Therefore, in the present study, we aimed to characterize the craniofacial abnormalities in the homozygous G608G mouse model.

## Materials and methods

Genotyping was performed using genomic DNA isolated from mouse tail cuts using the Red Extract-n-Amp tissue PCR kit (Sigma-Aldrich, St. Louis, MO, United States), then amplified by heminested PCR using forward (GenoCh4-F1, 5′-CAAACAAGTACATATCATAGGC-3′) and reverse (GenoCh4-R1, 5′-ATGATAGTGACAGGTATACGG-3′) primers external to the BAC insertion site on chromosome 4 and an additional reverse primer (GenoBAC-R2 5′-ATTCTAGTGGAGGGAGACAG-3′) located within the BAC sequence. Thermal cycling conditions included an initial denaturation at 95°C, then 35 cycles at 94°C, 58°C and 72°C for 45 s each. Genotype was detected by electrophoretic separation of 493bp (wild-type) and 233bp (transgene) amplification products.

All animal care was conducted at the National Institutes of Health (NIH) following Institutional Animal Care and Use Committee (IACUC) approval. Mice grew at a reduced rate for approximately 4 months and normally ate until 7 months of age, when attentiveness and activity began to decline. This caused progressive deterioration until eventual death before 8 months of age.

HGPS mice and age-matched WT littermates were imaged at the same time points of 2, 4, 6, and 8 months. The mean age at death for HGPS mice was 39.4 weeks (SD = 6.2 weeks). Each group consisted of 5 mice.

### Clinical computed tomography (CT) scans

**FIGURE 1:**
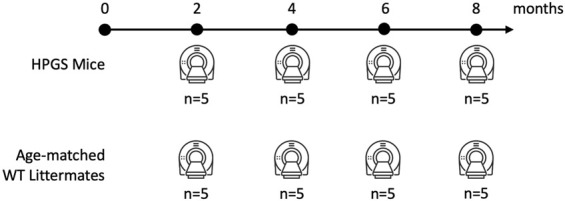
Illustration of the time points and the CT measurements for the study.

We obtained whole-body computed tomography (CT) scans (CT, Siemens, Inveon CT, Malvern, PA, United States) at 2, 4, 6, and 8 months of age (Figure 1). The animals were scanned using an exposure time of 325 ms, a voxel size of 100 μm, and a tube voltage of 80 kVp.

The 8-month assessment, which occurs as mice are nearing the endpoint, allows the most apparent pathology characterization due to the progressive nature of HGPS.

Whole-body computed tomography scans were used for image analysis with a Scanco 40 μCT scanner software (Scanco AG, Brüttisellen, Switzerland). To compute cranial bone volume and cortical thickness, 50 slices were contoured from the parietal to frontal regions of the skull as the volume of interest (VOI) for each sample (Figure 3A). The measurement we performed was not the conventional thresholded BV compared to the total volume of the region. Due to the low resolution of the images, we segmented out the region of interest and thresholded accordingly to include/highlight the whole area for a 3D volume measurement. The Bone volume presented is the entire volume of the analyzed area, the total volume of the cranial bone. We assessed whether the size of the cranial bone has changed by measuring a selected section for its total volume and thickness (distance from the coronal surface to the ventral surface of the skull).

### Three-dimensional (3D) craniofacial measurements

**FIGURE 2:**
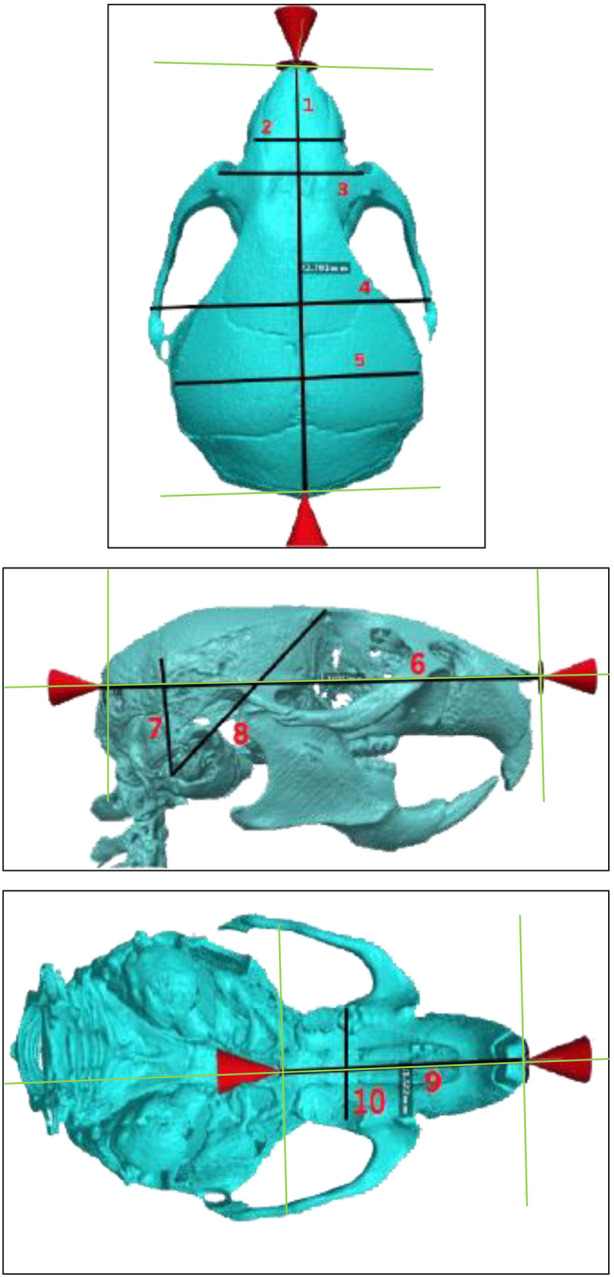
Superior (top), lateral (middle), and inferior (bottom) views of age-matched WT littermates. The specific cranial measurements 1–10 described in this study are marked on the image. Measurements Legend: Sagittal Cranial Length (1), Internasal Width I (2), Internasal Width II (3), Interzygomatic Distance (4), Bitemporal Distance (5), Sagittal Cranial (6), Posterior Cranial Height I (7), Posterior Cranial Height II (8), Palatine Length (9), Intermolar Distance (10).

The measurements chosen for this study were modeled based on the protocols designed by F. de Carlos *et al.* and H. Eimar, who performed 3D cephalometric measurements in mice (de Carlos et al., 2008; de Carlos et al., 2011; Eimar et al., 2016). In our experiment, 3D reconstruction was performed on CT data using the Simpleware ScanIP v7.0 software (Synopsys, Mountain View, United States). Skulls were segmented from whole-body scans, and precise measurements were performed directly on the 3D reconstructed surface. Ten different 3D cephalometric measurements were performed on the superior, inferior, and lateral aspects of the WT and HGPS mice skulls, as shown in Figure 2. These measurements include (Table 1):

**TABLE 1:** Craniofacial indices used in the study.

Craniofacial Index	Description
SCL	Sagittal Cranial Length
INWI	Internasal Width 1
IZD	Interzygomatic Distance
INWII	Internasal Width II
BTD	Bitemporal Distance
SC	Sagittal Cranial
PCHI	Posterior Cranial Height I
PCHII	Posterior Cranial Height II
PL	Palatine Length
ID	Intermolar Distance

#### Superior aspect

1. Sagittal Cranial Length: measured from the most anterior internasal suture point to the most posterior occipital point
2. Internasal Width I: measured between the right and left supraorbital foramens
3. Internasal Width II: distance measured 2 mm anterior to Internasal Width I.
4. Interzygomatic Distance: Measured between the zygomatic sutures on the left and right zygomatic arches
5. Bitemporal Distance: Lateral distance between the midpoints of the coronal suture and lambdoid suture on the superior temporal line

#### Lateral aspect

6. Sagittal Cranial: Measured between occipital and nasomaxillary point (anterior-most).
7. Posterior Cranial Height I: measured between tympanic bulla and the intersection of the lambdoid suture with the superior temporal line
8. Posterior Cranial Height II: Measured between tympanic bulla and the intersection of the coronal suture with the superior temporal line

#### Inferior aspect

9. Palatine Length: Measured between palatine (posterior-most) and interdental midpoints.
10. Intermolar Distance: Measured between the left and right molar fossas

### Statistical analysis

Normality was assessed with the Shapiro-Wilk test. A mixed model ANOVA test with repeated measures assessed differences between the groups (fixed effect) over the four time points (repeated variable). Post-hoc analyses with multiple comparisons were adjusted using the least significant difference and Bonferroni or Tukey methods. Estimated marginal means and standard deviations were used to report normally distributed outcomes. Two-tailed *p*-values less than 0.05 were considered significant. Statistical analyses were conducted using GraphPad Prism (Version 9, GraphPad Software, San Diego, California, United States.

## Results

### Bone volume and cortical thickness

**FIGURE 3:**
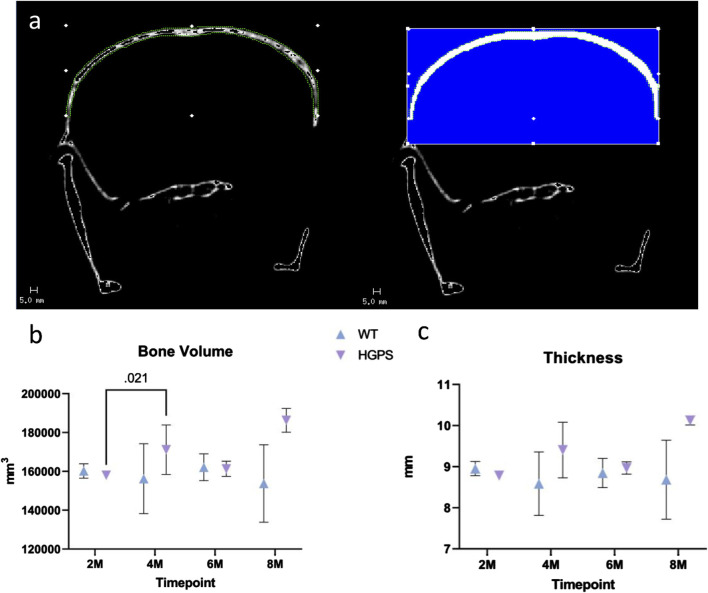
**(A)** Native CT image (left) with example contouring and VOI with blue area highlighting the applied threshold and the quantified region (right). **(B)** Mouse cranial bone volume measurements for 2, 4, 6, and 8-month-old animals. **(C)** Mouse cranial bone cortical thickness measurements for 2, 4, 6, and 8-month-old animals. Differences with *p* < 0.05 are marked for this Figure.

HGPS mice tend to have increased bone volume compared to the age-matched WT littermates (Figure 3B). Cranial bone volume in the age-matched WT littermates was variable yet relatively similar on average as the mice increased in age from 2 months to 8 months. In contrast, there was an increase in bone volume in HGPS mice as the mice age from 2 to 8 months. At 2 months of age, bone volume for HGPS mice was similar to age-matched WT littermates; however, by 4 months, bone volume was significantly increased compared to 2-month-old HGPS mice (*p* = 0.021). By 8 months of age, HGPS cranial bone volume was 21.2% greater than age-matched WT littermates (p = NS (0.17)); however, it did not reach significance.

The increased bone volume in HGPS mice may be attributed to alterations in cortical thickness compared to WT mice (Figure 3C). While cortical thickness remained relatively similar in WT mice as they aged, HGPS mouse cortical thickness increased from 2 to 8 months (p = NS (0.08)), resulting in a 16.6% greater thickness compared to the age-matched WT littermates (p = NS (0.224)), though it did not reach significance.

### Superior aspect

**FIGURE 4:**
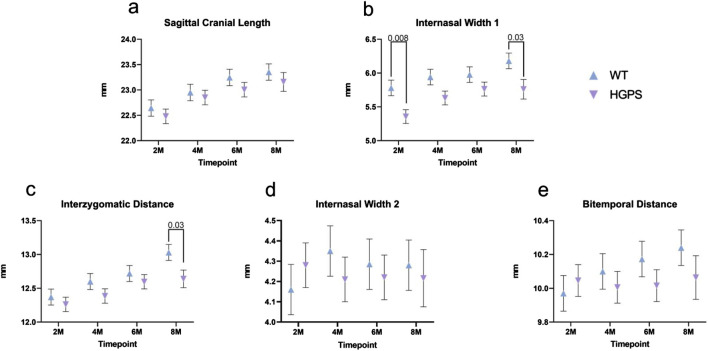
Three-dimensional measurements of the Superior Craniofacial aspect of 2, 4, 6, and 8-month-old animals. **(A)** Sagittal Cranial Length, **(B)** Internasal Width I, **(C)** Interzygomatic Distance, **(D)** Internasal Width II, and **(E)** Bitemporal Distance. Differences with *p* < 0.05 are marked for this Figure.

Sagittal Cranial Length (SCL): The animals in both groups had increased sagittal cranial length over the 8 months, as shown in Table 2 and Figure 4A. By 8 months, age-matched WT littermates exhibited a 3.15% (0.71 mm) increase in SCL (*p* < 0.001), while the HGPS mice reported a 3.02% increase (0.68 mm, (*p* < 0.001)).

**TABLE 2:** The reported craniofacial measurements for this study, with the *p* values reflecting differences between the two groups for each time point and measurement (*p* < 0.05 considered significant).

	Measurement	Group	2 months (mm)	*p* value	4 months (mm)	*p* value	6 months (mm)	*p* value	8 months (mm)	*p* value	% Change between 2 and 8 months
Superior aspect	Sagittal Cranial Length (SCL)	HGPS Mice	22.48		22.85		23.01		23.16		+3.02% (0.68 mm)
		Age-Matched WT Littermates	22.64	0.45	22.95	0.65	23.25	0.28	23.35	0.44	+3.15% (0.71 mm)
	Internasal Width 1 (INWI)	HGPS Mice	5.36		5.63		5.76		5.76		+7.56% (0.41 mm)
		Age-Matched WT Littermates	5.78	**0.008**	5.94	0.05	5.98	0.17	6.18	**0.03**	+6.94 (0.40 mm)
	Interzygomatic Distance (IZD)	HGPS Mice	12.26		12.39		12.60		12.64		+3.07% (0.38 mm)
		Age-Matched WT Littermates	12.37	0.52	12.60	0.18	12.72	0.44	13.03	**0.03**	+5.39% (0.67 mm)
	Internasal Width II (INWII)	HGPS Mice	4.28		4.21		4.22		4.22		−1.5% (0.06 mm)
		Age-Matched WT Littermates	4.16	0.46	4.35	0.40	4.29	0.70	4.28	0.75	+2.91% (0.12 mm)
	Bitemporal Distance (BTD)	HGPS Mice	10.05		10.01		10.02		10.06		+0.10% (0.01 mm)
		Age-Matched WT Littermates	9.97	0.61	10.10	0.51	10.17	0.27	10.24	0.29	+2.68% (0.27 mm)
Lateral view	Sagittal Cranial (SC)	HGPS Mice	4.68		4.34		4.39		4.41		−5.81% (0.27 mm)
		Age-Matched WT Littermates	4.53	0.33	4.61	0.08	4.85	**0.003**	4.69	0.15	+3.47% (0.16 mm)
	Posterior Cranial Height I (PCHI)	HGPS Mice	9.83		9.79		9.71		9.88		+0.51% (0.05 mm)
		Age-Matched WT Littermates	9.87	0.85	9.80	0.97	9.98	0.21	10.23	0.19	+3.65% (0.36 mm)
	Posterior Cranial Height II (PCHII)	HGPS Mice	22.81		23.18		23.30		23.45		+2.77% (0.63 mm)
		Age-Matched WT Littermates	23.13	0.22	23.34	0.54	23.68	0.15	23.85	0.16	+3.08% (0.71 mm)
Inferior	Palatine Length (PL)	HGPS Mice	11.41		11.55		11.46		11.10		−2.67% (−0.31 mm)
		Age-Matched WT Littermates	11.46	0.78	11.75	0.34	11.97	**0.01**	12.22	**<0.001**	+6.59% (0.75 mm)
	Intermolar Distance (ID)	HGPS Mice	4.86		4.89		4.98		4.86		0% (0 mm)
		Age-Matched WT Littermates	5.08	0.07	5.10	0.08	5.22	**0.04**	5.24	**0.01**	+3.29% (0.17 mm)

Internasal Width I (INWI): The groups exhibited an increase in internasal width over 8 months (Figure 4B). Starting at 2 months, the age-matched WT littermates exhibited an INWI measurement of 5.78 mm and an overall 6.94% (0.40 mm) increase by the end of the study period (*p* = 0.002) (8 months). On the other hand, HGPS mice started with a significantly lower INWI at 2 months (5.36 mm, *p* = 0.008) compared to age-matched WT littermates (5.78 mm). They maintained the lower INWI measurements at the end of the 8 months (5.76 mm, *p* = 0.009) compared to age-matched WT littermates. Significant differences were observed between the groups at the 4-month time point as well (5.94 mm age-matched WT littermates, 5.61 mm HGPS, *p* = 0.049).

Interzygomatic Distance (IZD): All groups exhibited an increase in interzygomatic distance over the 8-month study period (Figure 4C). Age-matched WT littermates exhibited a 5.39% (0.67 mm) increase in IZD between 2 and 8 months (*p* < 0.0001), while HGPS mice exhibited a lower increase (3.07%, 0.38 mm) during the same period. HGPS mice showed significantly lower IZD measurement at 8 months (12.64 mm, *p* = 0.001) than age-matched WT littermates (13.03 mm).

Internasal Width II (INWII): Age-matched WT littermates exhibited a 2.91% (0.12 mm) increase between 2 and 8 months (p = NS (0.23)), while the HGPS mice had a 1.50% decrease during the same period (0.06 mm, p = NS (0.61), Figure 4D). No significant differences were observed between the groups at any other time point.

Bitemporal Distance (BTD): Age-matched WT littermates exhibited a 2.68% (0.27 mm) increase in BTD between 2 and 8 months (*p* = 0.012), while HGPS mice showed a 0.10% increase during the same period (0.01 mm, p = NS (0.89) (Figure 4E). No significant differences were observed between the groups at any time point.

### Lateral aspect

**FIGURE 5:**
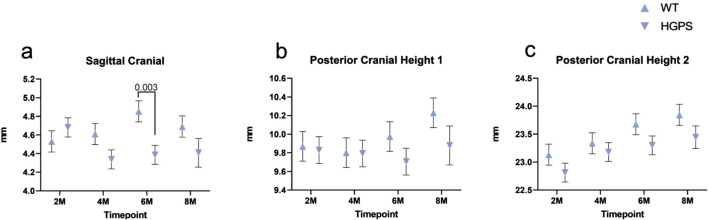
Three-dimensional measurements of the Lateral Craniofacial aspect of 2, 4, 6, and 8-month-old animals. **(A)** Sagittal Cranial, **(B)** Posterior Cranial Height I, **(C)** Posterior Cranial Height II. Differences with *p* < 0.05 are marked for this Figure.

Sagittal Cranial (SC): Age-matched WT littermates exhibited a 3.47% (0.16 mm) increase in SC between 2 and 8 months (p = NS (0.26)), while HGPS mice showed a 5.81% (0.27 mm) decrease during the same period (*p* = 0.021) (Figure 5A). Lower measurements in the HGPS mice became more evident at the 4 and 6-month CT scans, with significantly lower SC measurements at both follow-up times (*p* = 0.008 and *p* = 0.021, respectively). However, no differences were observed between the 2 and 8-month scans for the HGPS group (*p* = 0.113).

Posterior Cranial Height I (PCHI): Both groups exhibited relatively stable posterior cranial height I measurements between 2 and 6 weeks, with increases at 8 weeks (Figure 5B). Age-matched WT littermates exhibited a 3.65% (0.36 mm) increase in PCH1 between 2 and 8 months (*p* = 0.049), while HGPS mice showed a 0.51% (0.05 mm, p = NS (0.82)) increase during the same period. No significant differences were observed between the groups at any time point.

Posterior Cranial Height II (PCHII): Both groups exhibited an increase in posterior cranial height two over the 8-month study period (Figure 5C). Age-matched WT littermates exhibited a 3.08% (0.71 mm) increase in PCH2 between 2 and 8 months (*p* < 0.001), while HGPS mice showed a 2.77% increase (0.63 mm) during the same period (*p* < 0.001). Significant differences were observed between most time points in each group.

### Inferior aspect

**FIGURE 6:**
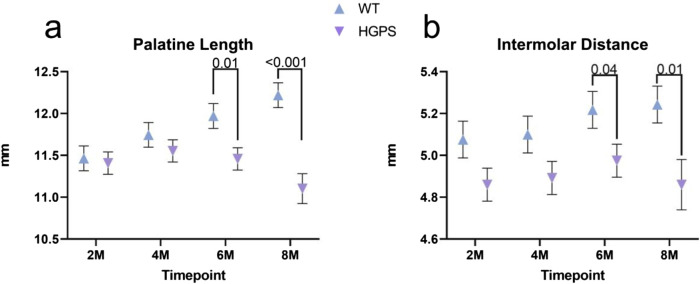
Three-dimensional measurements of the Inferior Craniofacial aspect of 2, 4, 6, and 8-month-old animals. **(A)** Palatine Length, **(B)** Intermolar Distance.

Palatine length (PL): Age-matched WT littermates exhibited an overall 6.59% (0.75 mm) increase in PL between 2 and 8 months (*p* < 0.0001), while HGPS mice showed a 2.67% (0.31 mm) decrease during the same period (p = NS (0.078)), with the decrease occurring predominantly between weeks 6 and 8 (Figure 6A). HGPS mice showed significantly lower PL measurements at 6 months (11.97 mm, *p* = 0.014) and 8 months (12.22 mm, *p* < 0.0001) compared to age-matched WT littermates (11.46 mm and 11.10 mm, respectively).

Intermolar Distance (ID): Age-matched WT littermates exhibited a 3.29% (0.17 mm) increase in intermolar distance from baseline (p = NS (0.132)), while HGPS mice showed little change in ID over time (p = NS (0.939)) (Figure 6B). More specifically, HGPS mice showed significantly lower ID measurements at 6 months (5.22 mm, *p* = 0.04) and 8 months (5.24 mm, *p* = 0.01) when compared to age-matched WT littermates (4.98 mm and 4.86 mm, respectively).

## Discussion

Using whole-body CT scans, we characterized craniofacial abnormalities in a homozygous G608G mouse model. A series of ten measurements at the skull’s superior, inferior, and lateral aspects were taken at the four time points (2, 4, 6, and 8 months) to determine several differences observed between HGPS and age-matched WT littermates at the end of the study. These include internasal width I, interzygomatic distance, sagittal cranial, palatine length, and intermolar distance. HGPS mice exhibited a significant increase in bone volume by 4 months compared to their earlier measurements (*p* = 0.021). By 8 months, their bone volume was 21.2% higher than those of WT mice, although not statistically significant (*p* = 0.170). The increased bone volume may be linked to greater cortical thickness, which was 16.6% thicker in HGPS mice at 8 months, although this also did not reach statistical significance (p = NS 0.224). Sagittal cranial length increased similarly in both groups (HGPS 3.02% vs. age-matched WT littermates 3.15%, p = NS 0.44). HGPS mice had a significantly smaller internasal width at two (*p* = 0.008) and 8 months (*p* = 0.03). Interzygomatic distance increased more in age-matched WT littermates (5.39% vs. HGPS 3.07%, *p* = 0.03). HGPS mice showed a significant decrease in sagittal cranial length by 6 months (*p* = 0.003). HGPS mice had significantly lower intermolar distances at 6 and 8 months (*p* = 0.04 and *p* = 0.01, respectively). These findings reflect the progressive nature of HGPS and are consistent with the observation that craniofacial abnormalities become more prominent with time. It should also be noted that INWI differed between HGPS mice and age-matched WT littermates at the earlier 2-month time point.

Our G608G homozygous mouse model showed significant decreases in various parameters (INWI, IZD, SC, PL, and ID) compared to age-matched WT mice, as mentioned above. However, we did not detect a consistent and significant reduction in *all* craniofacial measurements in HGPS mice. Thus, despite our more rigorous quantification of craniofacial features in the homozygous G608G mouse model, it remains difficult to effectively conclude an overall skull size reduction as reported in the homozygous G609G model by Osorio *et al.* In addition, most craniofacial abnormalities in our animal model without treatment were noted at later stages of disease progression (6–8 months follow-up). These findings are consistent with clinical descriptions of HGPS in humans, wherein abnormalities present around 2 years of age and worsen with time (Merideth et al., 2008). In addition, some of the most distinct craniofacial features of HGPS in humans include prominent eyes (due to orbital hypotelorism) and a narrow nasal bridge with a broad-tipped nose (Ullrich et al., 2012). These descriptions are accurately reflected in our HGPS G608G mice, which exhibited significantly decreased internasal width (INWI) compared to age-matched WT littermates; however, no observations were recorded about prominent eyes in this model. Moreover, our model is consistent with descriptions of maxillary hypoplasia in humans with HGPS Field (Hennekam, 2006) since our HGPS mice exhibited lower 8-month averages in palatine length (shorter snout) and intermolar distance (narrower face) compared to age-matched WT littermates.

Despite extensive clinical characterization of the musculoskeletal and craniofacial phenotypes of HGPS, relatively little is known about the molecular mechanisms underlying these abnormalities (Schmidt et al., 2012). Recent *in vitro* studies have linked the accumulation of progerin with altered cellular mechanisms that can explain the bone and cartilage tissue changes seen in HGPS disease (Schmidt et al., 2012; Gargiuli et al., 2018). More specifically, the osteoblast-specific expression of the HGPS mutation increases DNA damage and adversely affects the Wnt signaling pathway, impacting bone and cartilage configuration (Day et al., 2005). Defects in the Hippo pathway and *RUNX2* gene also contribute to musculoskeletal abnormalities; however, Wnt/beta-catenin derangements appear to be more central to developing the HGPS phenotype (Chen et al., 2008). Further, fibroblasts from the *LMNA* Δ9 progeria mouse model and hair follicle stem cells in ZMPSTE24-deficient mice have shown reduced *Wnt/β-catenin* signaling (Espada et al., 2008; Hernandez et al., 2010). This alteration leads to mesenchymal stem cell dysfunction (Harhouri et al., 2018) and abnormal expression of extracellular matrix genes with specific roles in skeletal and cartilage development (Day et al., 2005; Chen et al., 2008; Hernandez et al., 2010). In the teeth, these changes perturb dentin formation and cause severe dental abnormalities involving the incisors (Gargiuli et al., 2018). In addition, histopathologic analysis of a mouse model generated by Eriksson et al. demonstrated dramatic effects on bone microstructure, including hypocellular red bone marrow, widespread loss of osteocytes, and defects in mineralization (Eriksson et al., 2003). This mouse model also revealed fractured incisors, disturbed dentin formation, and the presence of secondary inflammatory reactions around the fracture sites. Bone biomarkers such as alkaline phosphatase, osteocalcin, and Collagen type I were also downregulated (Schmidt et al., 2012). More importantly, such disturbances in osteoblast differentiation were only observed after long-term expression of the HGPS mutation. Even though we did not assess for dental abnormalities of the incisors, blood levels of bone biomarkers, or perform a histopathological analysis of the craniofacial structures, our findings in the HGPS mice were also observed at later time points, where disease progression phenotypes are more evident.

Other laminopathy animal models have been generated to recapitulate bone microstructural abnormalities observed in HGPS patients. The first of these was the ZMPSTE24^−/−^ mouse model, which exhibits MSK abnormalities such as kyphosis, spontaneous bone fractures by 6–7 weeks of age, significant micrognathia, and a reduction of the zig-zag appearance of the cranial sutures (Bergo et al., 2002). More recently, the *Lmna* ^G609G/G609G^ homozygous mouse was generated, exhibiting joint immobility, skeletal deformities in the vertebral column (lordo-kyphosis), skulls with a marked size reduction, and smaller lower incisors (Osorio et al., 2012; Gargiuli et al., 2018). Our recent publication describing the bone phenotype in this mouse model may provide some insight into HGPS bone dysplasia (Cabral et al., 2023); although intramembranous and endochondral bone formation proceeds by different mechanisms, we demonstrated altered differentiation and functioning of long bone cell populations *in vitro* and *in vivo*. Specifically, we noted delayed maxillary, mandibular, and calvarial mineralization with increased craniofacial and mandibular cartilage content in newborn mice. Post-natal bone loss in these mice was associated with increased growth plate chondrocyte TUNEL staining, an adipogenic gene expression pattern with reduced *Wnt* signaling in osteoblasts and reduced osteoclastogenesis resulting from osteoblast-secreted factors (presumably OPG).

Detailed molecular studies characterizing gene expression and intracellular signaling pathways in *LMNA* ^G608G/G608G^ bone cell populations have not been published but are the subject of a current investigation. Alternative HGPS mouse models have consistently demonstrated a combination of decreased bone formation and resorption that is attributed to reduced numbers of osteoblasts, osteocytes, and osteoclasts *in vivo* (*Lmna* ^−/−^, *Lmna* ^HG/HG^, *Zmpste24* ^−/−^, and G608G tissue-specific tetop-LA^wt+^;Sp7-tTA^+^). Our prior analysis revealed significantly reduced bone turnover in *Lmna* ^G609G/G609G^ femora, which is attributed to defective osteoblast differentiation associated with an adipogenic gene expression signature and inhibition of osteoclastogenesis by osteoblast-secreted factors, presumably OPG (PMC1513052, 3081846, 130584, 19587107, 3460452). Thus, defects in bone remodeling are a common mechanism in the bone phenotype of HGPS mice.

Importantly, the development and growth of the cranium occur by two distinct processes. The first, endochondral ossification, requires deposition and replacement of a pre-existing cartilage model by bone and occurs mainly in the skull base. Endochondral bone formation, therefore, requires vascular invasion of the tissue, followed by the introduction of osteoclasts to resorb the pre-existing cartilage matrix. The second growth process, intramembranous ossification, requires neural crest-derived mesenchymal cells to directly differentiate into osteoblasts that deposit and mineralize the extracellular matrix, mainly in the cranium and facial skeleton. Intramembranous bones of the skull are joined by a fibrous matrix housing pluripotent progenitor cells that are continuously remodeled to allow for cranial enlargement. Furthermore, ossification of the sutures is concurrent with cessation of remodeling and cranial growth. We, therefore, highlight alterations to the normal processes of bone remodeling that are critical for the proper development of craniofacial skeletal architecture (Holmbeck and Szabova, 2006; Thilander, 1995) and that ours is the first study to link previously reported cellular defects in HGPS to craniofacial alterations demonstrated here.

One limitation of this study is the relatively small sample size (n = 5) of each transgenic mouse group. This low sample size may have increased measurement variability, which could account for the absence of significant differences in some craniofacial parameters. While our animal study has a sufficient sample size based on the degrees of freedom (Charan and Kantharia, 2013), a larger sample could more reliably predict differences in outcome parameters. Another limitation was the potential for unreliable results if different investigators carried out measurements. This was mitigated by assigning a single investigator to carry out these measurements consistently. Three-dimensional measurements were also pre-defined using the craniofacial landmarks to ensure precision. Previous studies have also found that 3D-CT volume rendering has small interobserver and intra-observer variation among measurements (Rocha Sdos et al., 2003; Cavalcanti et al., 2004).

In summary, the long bone phenotype in *Lmna* ^G609G/G609G^ mice is associated with a growth plate defect and low bone turnover condition that can be described as osteochondrodystrophy. Although these findings have not yet been confirmed in the G608G transgenic mouse model, our unpublished data has shown significantly reduced deposition of ECM and hydroxyapatite by G608G homozygous calvarial osteoblasts *in vitro* (manuscript in production). Thus, our work provides a unique opportunity for future studies in other animal models of HGPS to combine molecular, biomarker, and histopathological analyses with our proposed 3D cephalometric measurements. This combined strategy can contribute to a new understanding of potential disease mechanisms affecting the craniofacial structures in HGPS and improve and optimize therapeutic strategies. Most cephalometric measurements were decreased in the HGPS mice compared to age-matched WT littermates. These changes became more apparent as the animals aged, consistent with descriptions of disease progression in pediatric patients with progeria. Ultimately, this insight motivates future studies to test emerging therapeutic strategies on larger animal cohorts and ameliorate craniofacial abnormalities in humans with HGPS.

## Data Availability

The raw data supporting the conclusions of this article will be made available by the authors, without undue reservation.

## References

[B1] Bergo M. O. Gavino B. Ross J. Schmidt W. K. Hong C. Kendall L. V. (2002). Zmpste24 deficiency in mice causes spontaneous bone fractures, muscle weakness, and a prelamin A processing defect. Proc. Natl. Acad. Sci. U. S. A. 99, 13049–13054. 10.1073/pnas.192460799 12235369 PMC130584

[B2] Broers J. L. Ramaekers F. C. Bonne G. Yaou R. B. Hutchison C. J. (2006). Nuclear lamins: laminopathies and their role in premature ageing. Physiol. Rev. 86, 967–1008. 10.1152/physrev.00047.2005 16816143

[B3] Cabral W. A. Stephan C. Terajima M. Thaivalappil A. A. Blanchard O. Tavarez U. L. (2023). Bone dysplasia in Hutchinson-Gilford progeria syndrome is associated with dysregulated differentiation and function of bone cell populations. Aging Cell 22, e13903. 10.1111/acel.13903 37365004 PMC10497813

[B4] Cabral W. A. Tavarez U. L. Beeram I. Yeritsyan D. Boku Y. D. Eckhaus M. A. (2021). Genetic reduction of mTOR extends lifespan in a mouse model of Hutchinson-Gilford Progeria syndrome. Aging Cell 20, e13457. 10.1111/acel.13457 34453483 PMC8441492

[B5] Capell B. C. Olive M. Erdos M. R. Cao K. Faddah D. A. Tavarez U. L. (2008). A farnesyltransferase inhibitor prevents both the onset and late progression of cardiovascular disease in a progeria mouse model. Proc. Natl. Acad. Sci. U. S. A. 105, 15902–15907. 10.1073/pnas.0807840105 18838683 PMC2562418

[B6] Cavalcanti M. G. Rocha S. S. Vannier M. W. (2004). Craniofacial measurements based on 3D-CT volume rendering: implications for clinical applications. Dentomaxillofac Radiol. 33, 170–176. 10.1259/dmfr/13603271 15371317

[B7] Charan J. Kantharia N. D. (2013). How to calculate sample size in animal studies? J. Pharmacol. Pharmacother. 4, 303–306. 10.4103/0976-500X.119726 24250214 PMC3826013

[B8] Chen M. Zhu M. Awad H. Li T. F. Sheu T. J. Boyce B. F. (2008). Inhibition of beta-catenin signaling causes defects in postnatal cartilage development. J. Cell Sci. 121, 1455–1465. 10.1242/jcs.020362 18397998 PMC2636704

[B9] Day T. F. Guo X. Garrett-Beal L. Yang Y. (2005). Wnt/beta-catenin signaling in mesenchymal progenitors controls osteoblast and chondrocyte differentiation during vertebrate skeletogenesis. Dev. Cell 8, 739–750. 10.1016/j.devcel.2005.03.016 15866164

[B10] De Carlos F. Noval I. Vega J. (2011). 3D-µCT cephalometric measurements in mice. Comput. Tomogr. Appl. 10, 5772. 10.5772/24234

[B11] De Carlos F. Varela I. Germana A. Montalbano G. Freije J. M. Vega J. A. (2008). Microcephalia with mandibular and dental dysplasia in adult Zmpste24-deficient mice. J. Anat. 213, 509–519. 10.1111/j.1469-7580.2008.00970.x 19014358 PMC2667545

[B12] De Sandre-Giovannoli A. Bernard R. Cau P. Navarro C. Amiel J. Boccaccio I. (2003). Lamin a truncation in Hutchinson-Gilford progeria. Science 300, 2055. 10.1126/science.1084125 12702809

[B13] Eimar H. Tamimi F. Retrouvey J. M. Rauch F. Aubin J. E. Mckee M. D. (2016). Craniofacial and dental defects in the Col1a1Jrt/+ mouse model of osteogenesis imperfecta. J. Dent. Res. 95, 761–768. 10.1177/0022034516637045 26951553

[B14] Eriksson M. Brown W. T. Gordon L. B. Glynn M. W. Singer J. Scott L. (2003). Recurrent *de novo* point mutations in lamin A cause Hutchinson-Gilford progeria syndrome. Nature 423, 293–298. 10.1038/nature01629 12714972 PMC10540076

[B15] Espada J. Varela I. Flores I. Ugalde A. P. Cadinanos J. Pendas A. M. (2008). Nuclear envelope defects cause stem cell dysfunction in premature-aging mice. J. Cell Biol. 181, 27–35. 10.1083/jcb.200801096 18378773 PMC2287278

[B16] Gargiuli C. Schena E. Mattioli E. Columbaro M. D'apice M. R. Novelli G. (2018). Lamins and bone disorders: current understanding and perspectives. Oncotarget 9, 22817–22831. 10.18632/oncotarget.25071 29854317 PMC5978267

[B17] Goldman R. D. Shumaker D. K. Erdos M. R. Eriksson M. Goldman A. E. Gordon L. B. (2004). Accumulation of mutant lamin A causes progressive changes in nuclear architecture in Hutchinson-Gilford progeria syndrome. Proc. Natl. Acad. Sci. U. S. A. 101, 8963–8968. 10.1073/pnas.0402943101 15184648 PMC428455

[B18] Harhouri K. Frankel D. Bartoli C. Roll P. De Sandre-Giovannoli A. Levy N. (2018). An overview of treatment strategies for Hutchinson-Gilford Progeria syndrome. Nucleus 9, 246–257. 10.1080/19491034.2018.1460045 29619863 PMC5973194

[B19] Hennekam R. C. (2006). Hutchinson-Gilford progeria syndrome: review of the phenotype. Am. J. Med. Genet. A 140, 2603–2624. 10.1002/ajmg.a.31346 16838330

[B20] Hernandez L. Roux K. J. Wong E. S. Mounkes L. C. Mutalif R. Navasankari R. (2010). Functional coupling between the extracellular matrix and nuclear lamina by Wnt signaling in progeria. Dev. Cell 19, 413–425. 10.1016/j.devcel.2010.08.013 20833363 PMC2953243

[B21] Holmbeck K. Szabova L. (2006). Aspects of extracellular matrix remodeling in development and disease. Birth Defects Res. C Embryo Today 78, 11–23. 10.1002/bdrc.20064 16622846

[B22] Merideth M. A. Gordon L. B. Clauss S. Sachdev V. Smith A. C. Perry M. B. (2008). Phenotype and course of Hutchinson-Gilford progeria syndrome. N. Engl. J. Med. 358, 592–604. 10.1056/NEJMoa0706898 18256394 PMC2940940

[B23] Osorio F. G. Barcena C. Soria-Valles C. Ramsay A. J. De Carlos F. Cobo J. (2012). Nuclear lamina defects cause ATM-dependent NF-κB activation and link accelerated aging to a systemic inflammatory response. Genes Dev. 26, 2311–2324. 10.1101/gad.197954.112 23019125 PMC3475803

[B24] Rocha Sdos S. Ramos D. L. Cavalcanti Mde G. (2003). Applicability of 3D-CT facial reconstruction for forensic individual identification. Pesqui. Odontol. Bras. 17, 24–28. 10.1590/s1517-74912003000100005 12908055

[B25] Schmidt E. Nilsson O. Koskela A. Tuukkanen J. Ohlsson C. Rozell B. (2012). Expression of the Hutchinson-Gilford progeria mutation during osteoblast development results in loss of osteocytes, irregular mineralization, and poor biomechanical properties. J. Biol. Chem. 287, 33512–33522. 10.1074/jbc.M112.366450 22893709 PMC3460452

[B26] Thilander B. (1995). Basic mechanisms in craniofacial growth. Acta Odontol. Scand. 53, 144–151. 10.3109/00016359509005964 7572089

[B27] Ullrich N. J. Silvera V. M. Campbell S. E. Gordon L. B. (2012). Craniofacial abnormalities in Hutchinson-Gilford progeria syndrome. AJNR Am. J. Neuroradiol. 33, 1512–1518. 10.3174/ajnr.A3088 22460337 PMC7966543

[B28] Varga R. Eriksson M. Erdos M. R. Olive M. Harten I. Kolodgie F. (2006). Progressive vascular smooth muscle cell defects in a mouse model of Hutchinson-Gilford progeria syndrome. Proc. Natl. Acad. Sci. U. S. A. 103, 3250–3255. 10.1073/pnas.0600012103 16492728 PMC1413943

